# Genome-wide identification and evolutionary analyses of bZIP transcription factors in wheat and its relatives and expression profiles of anther development related *TabZIP* genes

**DOI:** 10.1186/s12864-015-2196-7

**Published:** 2015-11-18

**Authors:** Xueyin Li, Shiqing Gao, Yimiao Tang, Lei Li, Fengjie Zhang, Biane Feng, Zhaofeng Fang, Lingjian Ma, Changping Zhao

**Affiliations:** Beijing Engineering Research Center for Hybrid Wheat, Beijing Academy of Agriculture and Forestry Sciences, Beijing, 100097 China; College of Agronomy, Northwest A & F University, Yangling, 712100 China; State Key Laboratory of Protein and Plant Gene Research, Peking-Tsinghua Center for Life Sciences, College of Life Sciences, Peking University, Beijing, 100871 China; College of Agriculture, Shanxi Agricultural University, Taigu, 030800 China

**Keywords:** Basic leucine zipper (bZIP), Common wheat, Orthology analyses, Gene expression, Anther development, Over-dominance

## Abstract

**Background:**

Among the largest and most diverse transcription factor families in plants, basic leucine zipper (bZIP) family participate in regulating various processes, including floral induction and development, stress and hormone signaling, photomorphogenesis, seed maturation and germination, and pathogen defense. Although common wheat (*Triticum aestivum* L.) is one of the most widely cultivated and consumed food crops in the world, there is no comprehensive analysis of bZIPs in wheat, especially those involved in anther development. Previous studies have demonstrated wheat, *T. urartu*, *Ae. tauschii*, barley and *Brachypodium* are evolutionarily close in Gramineae family, however, the real evolutionary relationship still remains mysterious.

**Results:**

In this study, 187 bZIP family genes were comprehensively identified from current wheat genome. 98, 96 and 107 members of bZIP family were also identified from the genomes of *T.urartu*, *Ae.tauschii* and barley, respectively. Orthology analyses suggested 69.4 % of TubZIPs were orthologous to 68.8 % of AetbZIPs and wheat had many more in-paralogs in the bZIP family than its relatives. It was deduced wheat had a closer phylogenetic relationship with barley and *Brachypodium* than *T.urartu* and *Ae.tauschii*. bZIP proteins in wheat, *T.urartu* and *Ae.tauschii* were divided into 14 subgroups based on phylogenetic analyses. Using Affymetrix microarray data, 48 differentially expressed *TabZIP* genes were identified to be related to anther development from comparison between the male sterility line and the restorer line. Genes with close evolutionary relationship tended to share similar gene structures. 15 of 23 selected *TabZIP* genes contained LTR elements in their promoter regions. Expression of 21 among these 23 *TabZIP* genes were obviously responsive to low temperature. These 23 *TabZIP* genes all exhibited distinct tissue-specific expression pattern. Among them, 11 *TabZIP* genes were predominantly expressed in anther and most of them showed over-dominance expression mode in the cross combination TY806 × BS366.

**Conclusions:**

The genome-wide identification provided an overall insight of *bZIP* gene family in wheat and its relatives. The evolutionary relationship of wheat and its relatives was proposed based on orthology analyses. Microarray and expression analyses suggested the potential involvement of *bZIP* genes in anther development and facilitated selection of anther development related gene for further functional characterization.

**Electronic supplementary material:**

The online version of this article (doi:10.1186/s12864-015-2196-7) contains supplementary material, which is available to authorized users.

## Background

Transcription factors play vital roles in almost all plant biological processes [1]. Among the largest and most diverse dimerizing transcription factor families, the basic leucine zipper (bZIP) family of proteins in plants participates in regulating various processes including floral induction and development, stress and hormone signaling, photomorphogenesis, seed maturation and germination, and pathogen defense [1, 2].

The bZIP proteins characteristically possess a bZIP domain composed of a basic region and a leucine zipper [3]. The highly conserved basic region consists of approximately 16 amino acid residues that contain an invariant N-x7-R/K motif and that are responsible for sequence-specific DNA binding [1, 2]. The less conserved leucine zipper is composed of a heptad repeat of Leu or other bulky hydrophobic amino acids (i.e., Ile, Val, Phe or Met) positioned exactly nine amino acids toward the C-terminus to form an amphipathic helix, which confers homo- or hetero-dimerization specificity [2]. Plant bZIP proteins preferentially bind to DNA sequences contain an ACGT core, especially like G-box (CACGTG), C-box (GACGTC) and A-box (TACGTA) [4, 5].

Members of the bZIP transcription factor family have been comprehensively identified or predicted in several plant species [1, 2, 6–12]. In the model plants Arabidopsis and rice, 76 and 94 distinct members of the bZIP family have been identified and analyzed, respectively [1, 2]. bZIP family members of Arabidopsis have been classified into ten groups based on a similar basic region and additional conserved motifs outside the bZIP domain [1]. OsbZIP proteins are divided into 11 groups based on their putative DNA-binding specificity and dimerization properties [2].

Until now, some bZIP transcription factors have been reported to function in the anther development. In Arabidopsis, *AtbZIP1* overexpression is detrimental to pollen development and *AtbZIP1* is thought to be closely related to water movement during anther development, when anthers first absorb water for growth but dehydrate before dehiscence [13–15]. *AtbZIP34* controls pollen wall patterning and several metabolic pathways in developing pollen [16, 17]. Among the ten members of TGACG (TGA) motif-binding proteins composing a distinct subgroup in the bZIP family of Arabidopsis, *TGA 9* and *TGA10* have been reported to play a role in anther patterning and dehiscence. TGA9/10, together with ROXY1/2, positively regulate a common set of genes that contribute to the development of the tapetal, which provides nutrients and secretes enzymes and structural components of the pollen coat [1, 18]. In rice, *OsABI5* is highly expressed in mature pollen and suppression of *OsABI5* expression in transgenic rice lines causes abnormal mature pollen development and low fertility [19, 20]. However, among the largest and most diverse transcription factor families, bZIP gene family have not been systematically identified from the wheat genome. And, to date, few wheat bZIP transcription factors have been identified to be involved in anther development.

The hexaploid bread wheat (*Triticum aestivum*; 2n = 6x = 42; AABBDD) derived from two hybridizations in the evolution history between three gramineous ancestors. Wheat arose as a result of hybridization between the cultivated tetraploid emmer wheat (*T.dicoccoides*, AABB) and diploid goat grass (*Ae.tauschii*, DD) approximately 8,000 years ago. The three sub-genomes of wheat evolved from *T.urartu* (A sub-genome), from a species that might be from the section *Sitopsis* (B sub-genome), and from *Ae.tauschii* (D sub-genome), respectively [21]. Wheat is one of the most widely cultivated and consumed food crops in the world [22, 23]. Recently, an ordered draft sequence of the 17Gb hexaploid bread wheat genome has been generated by sequencing isolated chromosome arms and major efforts have been undertaken worldwide to sequence and annotate the wheat genome [21–28].

Wheat thermosensitive genic male sterile (TGMS) lines such as BS366 are of great importance for the utilization of heterosis in wheat breeding [29]. When exposed to low temperature conditions, BS366 becomes sterile, thus allowing the large-scale production of F1 hybrids via crossing with wheat restorer lines. The male sterility phenotype is heritable in wheat TGMS lines and controlled strictly by temperature [29, 30]. Previous studies have demonstrated that male fertility in BS366 is controlled by temperature during the period from the pollen mother cell (PMC) stage to the meiosis stage [31]. The two-line cross combinations of BS-series are our exclusive plant materials for wheat breeding and several of them have been widely used in wheat breeding and production. As important parental materials used in two-line combined breeding, BS366 (male sterility line) and TY806 (restorer line) generate a fertility-restored F1 hybrid. Thus, the cross combination of BS366, TY806 and their hybrid F1 is the excellent candidate for studying anther development in wheat.

In this study, we report the identification of bZIP family genes in the current genomes of wheat and its relatives, including *Triticum urartu*, *Aegilops tauschii* and *Hordeum vulgare*. Then, we have identified the orthologs and in-paralogs between each pair of wheat and its relatives. Further, we have proposed the evolutionary relationship among wheat and its relatives based on the orthology analyses of bZIP family. bZIP transcription factors in wheat, *T.urartu* and *Ae.tauschii* have been classified on the basis of phylogenetic analyses. 23 anther development related *bZIP* genes are selected from differentially expressed genes by microarray data analyses. Their gene structure and *cis*-acting elements have been also analyzed. Expression analyses have been performed to reveal their expression profile in response to low temperature and tissue-specific expression pattern in wheat male sterility line BS366, restorer line TY806 and their F1 hybrid and an emphasis is laid on expression in anther. Our results deepen the understanding of evolutionary relationship of wheat and its relatives and provide a prospective for biological involvement of bZIP family genes in anther development and heterosis of wheat.

## Results

### Identification of bZIP transcription factors in wheat, *T. urartu*, *Ae. tauschii* and barley

In the Gramineae family, except for *T.urartu* and *Ae.tauschii*, barley and *Brachypodium* are also close relatives of wheat. The evolutionary relationships between rice, *Brachypodium*, barley and wheat have been speculated based on the mean synonymous substitution rates (Ks) of orthologous gene pairs [32]. To identify bZIP transcription factors in wheat, *T. urartu*, *Ae. tauschii*, barley and *Brachypodium*, a hidden Markov model (HMM) search was performed using the HMM profiles of the bZIP domain (Pfam accession No.: PF00170 and PF07716) as queries against the respective protein databases. As a result, the amino acid sequences of 100 possible bZIP transcription factors were obtained using a Perl program from HMM search hits against the *T. urartu* and *Ae. tauschii* protein databases, respectively. Among these sequences, three contig sequences were removed from the analysis. Subsequently, two *T. urartu* proteins and one *Ae. tauschii* protein were discarded because they lacked the typical bZIP domain, finally resulting in the identification of 98 *T. urartu* and 96 *Ae. tauschii* bZIP transcription factors. Similarly, 182 non-redundant bZIP proteins were identified in the wheat genome and named. All these wheat *bZIP* genes were designated as *TabZIP* genes and given a number designation from 1 to 182 as an unique identifier as proposed for bZIP transcription factors in Arabidopsis [1]. The nomenclature was based on the positions of these genes on the wheat chromosomes, from the short arm to the long arm and in the order of 1A to 7A, 1B to 7B and 1D to 7D. Additionally, several *TabZIP* genes had been identified in previous studies [33–39]. *TaABF1* [33] and *TabZIP60* [34] were identical to *TabZIP23* and *TabZIP51* in our 182 wheat bZIP family members, respectively. *Wlip19a*, *Wlip19b* and *Wlip19d* (three homoeologous loci of *WLIP19* on chromosomes 1A, 1B, and 1D) [35] were identical to *TabZIP2*, *TabZIP59* and *TabZIP123*, respectively. *TaOBF1b* and *TaOBF1d* [35] were identical to *TabZIP98* and *TabZIP149*. However, *TaOBF1a* [35], *WABI5* [36], *TaABP1* [37], *TaABI5* [38] and *TaABL1* [39] was not within 182 bZIP family members identified by us. Finally, these 5 *TabZIP* genes were added to wheat *bZIP* gene family. So a total of 187 wheat *bZIP* genes were identified from the current wheat genome. Similarly, the 98 *T. urartu* and 96 *Ae. tauschii bZIP* genes were designated as *TubZIP1-98* and *AetbZIP1-96*, respectively. Furthermore, 107 bZIP proteins were also seperated from the barley genome. The *Brachypodium* genome had been repoted to contain 96 *bZIP* genes [12]. The nomenclature and chromosomal location of all *bZIP* genes are shown in Additional file 1: Table S1.

### Orthology and in-paralogy of *T. urartu* and *Ae. tauschii* bZIP proteins

Orthologous genes in different species originate from a single gene in the last common ancestor of these species and are therefore likely to share the same function [40, 41]. Homologs deriving from gene duplications are called paralogs. Because gene duplication events take place both before and after speciation, paralogy can also exist between genes in different species. Paralogs that originate following a gene duplication after speciation are termed as “in-paralogs” [42].

**Fig. 1:**
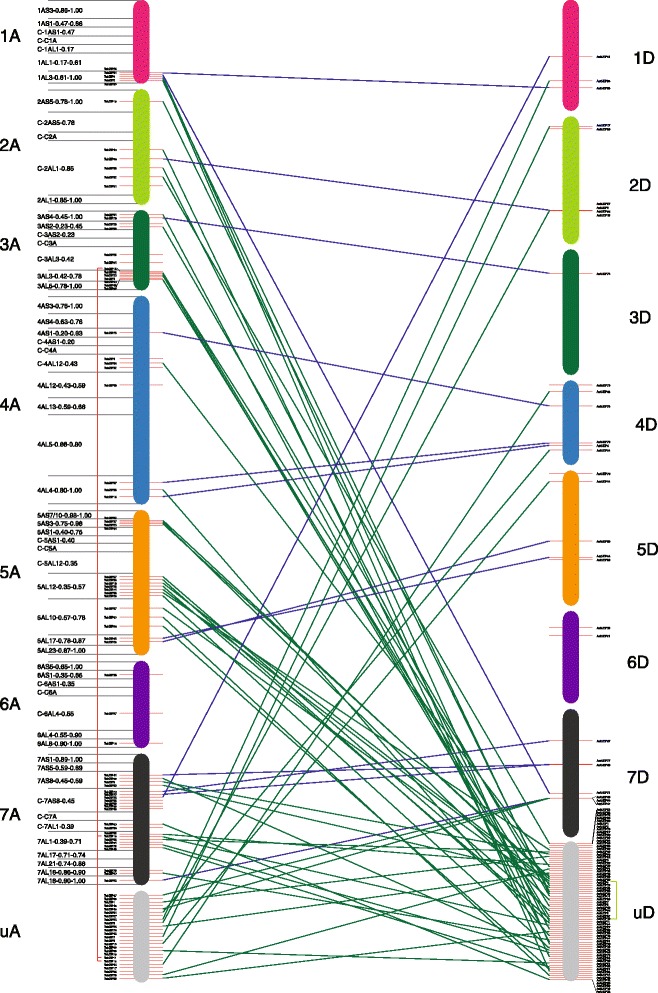
Orthologs between *T.urartu* and *Ae.tauschii* and in-paralogs within each organism of them. Each chromosome of *T.urartu* and *Ae.tauschii* was figured by actual proportion to others. The chromosomal scales of *T.urartu* was different from *Ae.tauschii*. The top and bottom of each stick standing for one chromosome were starting point and terminal point of the chromosome, respectively. The deletion bin maps of *T. urartu* 1A–7A chromosomes were noted on the left side, and *bZIP* genes in each deletion bin were averagely distributed. ‘uA’ and ‘uD’ mean genome regions not located to specific chromosomes, and genes in these regions were averagely distributed, thus the positions in the figure do not stand for the real locations in the genome

*T. urartu* and *Ae. tauschii* are the progenitors of the A and D genomes of hexaploid wheat (2 N = 6X = 42; AABBDD), respectively. A preliminary analyses have revealed that *T. urartu* and *Ae. tauschii* are evolutionarily close. To gain a better understanding of the evolutionary relationship of *bZIP* genes, InParanoid 7 (Center for Genomics and Bioinformatics, Stockholm, Sweden) was used to identify orthologs and in-paralogs between *T. urartu* and *Ae. tauschii* (Fig. 1). Among the bZIP proteins from *T. urartu* and *Ae. tauschii*, 65 groups of orthologs that were composed of 68 (69.4 %) *T. urartu* and 66 (68.8 %) *Ae. tauschii* proteins were detected using InParanoid 7 (Additional file 2: Figure S1). The largest group of orthologs contained 4 *T. urartu* proteins and 2 *Ae. tauschii* proteins and showed a many-to-many type of orthology. In this case, *TubZIP18*, *TubZIP11*, *TubZIP12* and *TubZIP15*, as in-paralogs from the same cluster, were co-orthologous to *AetbZIP10* and *AetbZIP5*. According to the clustering algorithm [40], *TubZIP11*, *TubZIP12* and *TubZIP15* originated from the gene duplication of *TubZIP18*, while *AetbZIP5* was derived from *AetbZIP10*. The remaining groups of orthologs between *T. urartu* and *Ae. tauschii* represented straightforward one-to-one orthology. Results showed that there were few in-paralogs in whether *T. urartu* or *Ae. tauschii* for *bZIP* gene family. Furthermore, bZIP family members in *T. urartu* shared high similarity in protein sequence with those in *Ae. tauschii*, indicating gene functions of bZIP family might remain conserved between *T. urartu* and *Ae. tauschii*, which was consistent with the study by Li [43].

### Evolutionary relationship of wheat, *T. urartu*, *Ae. tauschii*, barley and *Brachypodium*

**Fig. 2:**
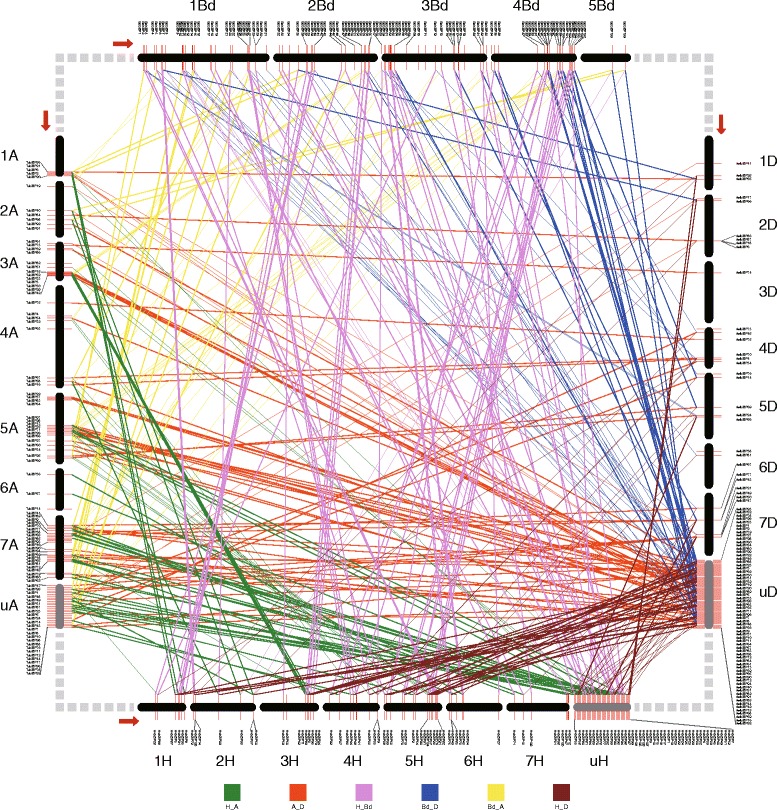
Orthologs between *T. urartu* (A), *Ae. tauschii* (D), *B. distachyon* (Bd) and *H. vulgare* (H). “1A”-“7A”, “1D”-“7D”, “1 Bd”-“5 Bd” and “1H”-“7H” represented chromosomes of *T. urartu*, *Ae. tauschii*, *B. distachyon* and *H. vulgare*,respectively. The chromosomes for the same species were drawn to scale based on actual proportion, but chromosomes of different species were figured by different scales. The direction for each chromosome from starting point to terminal point was indicated by the red arrow. ‘uA’, ‘uD’ and ‘uHv’ mean genome regions not located to specific chromosomes, and genes in these regions were averagely distributed, thus the positions in the figure do not stand for the real locations in the genome. The light-grey dashed rectangles in each corner just mean linkers between species. Ortholog pairs for the same pairs of species were linked by straight lines of the same color as indicated by the colored block at the bottom. For example, each pair of orthologs between *T. urantu* and barley were linked with a green straight line

To further explore the evolutionary relationship of bZIP family genes among these five evolutionarily close species, we identified orthologs and in-paralogs between each pair of these five species (Fig. 2 and Additional file 1: Table S2) using InParanoid 7, which allowed us to define the evolutionary point of the orthology precisely. To provide a simple explanation, the basic principle was that the number of orthologous groups between closely related species was expected to be greater than that between distantly related species [40]. The number of orthologs in each organism clustered with one other genome was summarized in Table 1. Notably, the table was not a symmetrical table because the gene duplication frequency in organism A generally differed from that in organism B since the speciation of organism A and B. Based on Table 1, wheat had a high average ortholog group size of 1.702 such that every *TabZIP* gene had an average of 0.702 paralogs. The average ortholog group size in *T. urartu*, *Ae. tauschii*, Barley and *Brachypodium* were found to be significantly smaller than that in wheat, which was consistent with the observation that these four genomes all had relatively fewer in-paralogs compared to the wheat genome [21–23, 32, 44]. Additional file 1: Table S2 also showed the intuitive observation that wheat had many more in-paralogs of *bZIP* genes than the other genomes.

**Table 1:** Total number of orthologs identified by In-paranoid 7 software

Species	Wheat	*T.urartu*	*Ae.tauschii*	Barley	*Brachypodium*	*bZIP* genes	Average size of ortholog groups (number of in-paralogs)
Wheat	-	76/49	78/54	110/64	113/54	182	1.702
*T.urartu*	52/49	-	68/65	63/60	50/45	98	1.067
*Ae.tauschii*	54/54	66/65	-	61/61	51/49	96	1.014
Barley	70/64	65/60	69/61	-	78/72	107	1.098
*Brachypodium*	55/54	53/45	54/49	72/72	-	88	1.075

The number of orthologs in an organism (y-axis) when clustered with another genome (x-axis) was shown on the left of the slash while the number on the right of the slash referred to the number of ortholog groups between two species. Thus, 76 wheat genes had orthologs in *T.urartu*, which were orthologous to a total of 52 *T.urartu* genes and wheat and *T.urartu* had totally 49 ortholog groups. ‘*bZIP* genes’ referred to the total number of *bZIP* genes in one organism. Only one protein for each *bZIP* gene in five genomes was used in In-paranoid clustering. ‘Average size of ortholog groups’ referred to the average number of in-paralogs in one ortholog group for an organism. For wheat, average size of ortholog group: 1.702 = (76/49 + 78/54 + 110/64 + 113/54)/4. Notably, Table 1 was not a symmetrical table, since gene duplication frequency in organism ‘A’ generally differed from that in organism ‘B’ since speciation of organism ‘A’ and ‘B’ and thus the number of ‘A’ genes having orthologs in organism ‘B’ was unequal to the number of ‘B’ genes having orthologs in organism ‘A’.

**Fig. 3:**
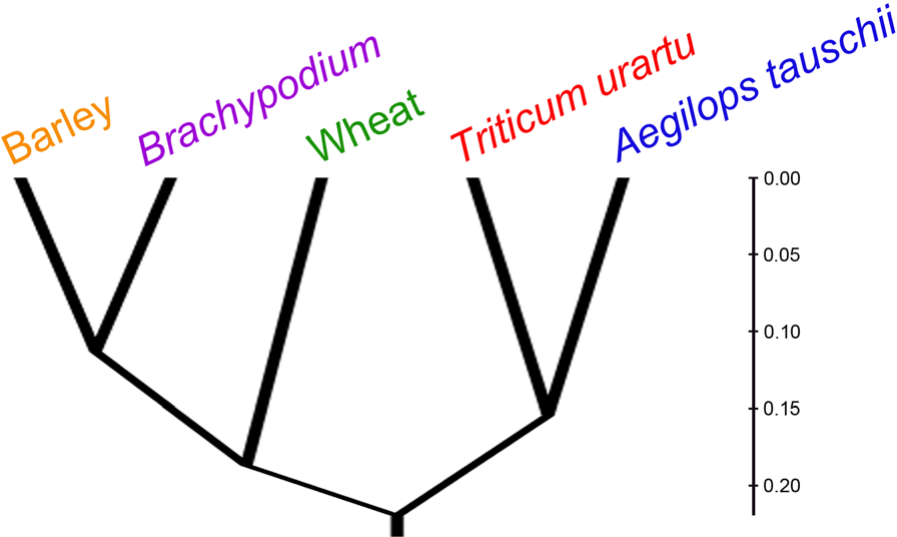
Orthophylogram of wheat and its four close relatives. This UPGMA tree was based on the average orthology distance between species

To reflect the level of orthology of the bZIP family genes between these five species, we constructed a phylogenetic tree termed an orthophylogram [41]. The orthology distance from species A to B (dAB) was calculated using the following formula [41], and the average orthology distance (dAB + dBA)/2 was then used to generate a UPGMA tree [41], as shown in Fig. 3.

$$ dAB=\frac{proteins\_A- proteins\_A\_ orthologous\_to\_B}{proteins\_A} $$

This orthophylogram demonstrated quantitatively the level of orthology of the bZIP gene family between different species. It was observed from the orthophylogram that wheat had a closer phylogenetic relationship with barley and *Brachypodium* than *T. uratu* and *Ae. tauschii*.

In plants, the structure and function of most of the *bZIP* genes most likely remained conserved during angiosperm evolution [2], thus, the results of our study provided deeper insight into the possible evolutionary relationship of five close relatives, namely, wheat, *T. urartu*, *Ae. tauschii*, barley and *B. distachyon*.

### Phylogenetic analysis of *bZIP* genes

**Fig. 4:**
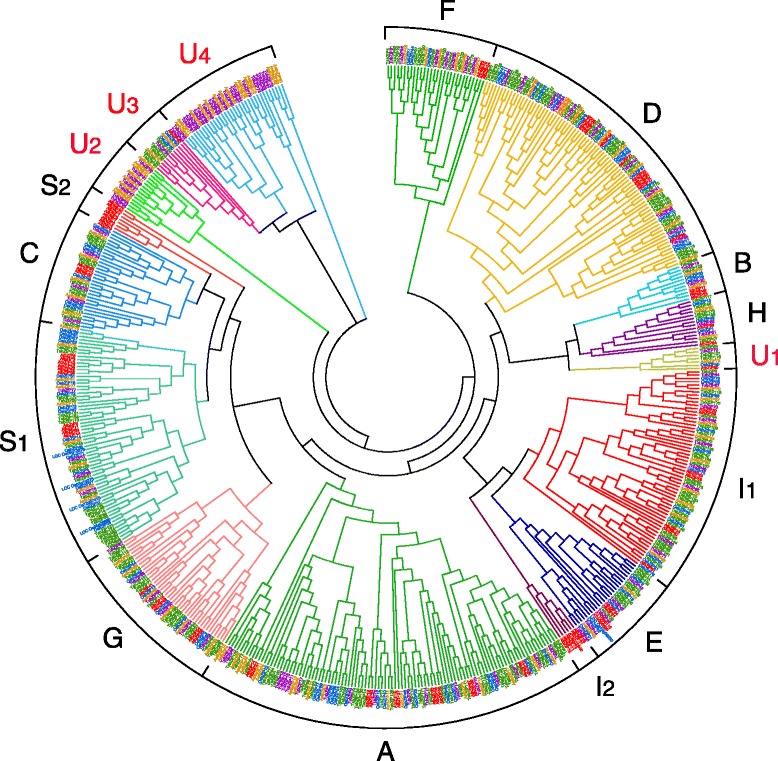
Phylogenetic relationship among TabZIP, TubZIP, AetbZIP, AtbZIP and OsbZIP proteins. The phylogenetic tree was based on the sequence alignment of the bZIP proteins. The unrooted tree was generated using ClustalX 2.1 program and displayed by MEGA 5.0. TabZIP, TubZIP and AetbZIP proteins were mainly classified into 10 subgroups (**a**, **b**, **c**, **d**, **e**, **f**, **g**, **h**, **i** and **s**) and 4 clades based on our phylogenetic analysis and Jakoby’s classification method of Arabidopsis bZIP family. With the newly identified bZIP members, S subgroup were divided into two clades S1 and S2 by the members in C subgroup and the E subgroup seprated protein members in the I subgroup into two clades I1 and I2. U1-U4 were 4 unnamed clades for lack of grouped AtbZIPs

The phylogenetic analysis was performed with all identified TabZIP, TubZIP and AetbZIP proteins as well as 76 Arabidopsis and 94 rice bZIP family members (Fig. 4). The TabZIP, TubZIP and AetbZIP proteins were classified primarily into 10 subgroups, which were named A, B, C, D, E, F, G, H, I and S according to a previously described classification method used for Arabidopsis bZIP family [1]. With the newly identified bZIP sequences, we found that subgroups S and I could be further divided into S1 and S2 and into I1 and I2, respectively. U1–U4 represented four previously unnamed clades, including AtbZIP60 and AtbZIP62, which did not fit into any of the 10 known subgroups [1]. Another ungrouped Arabidopsis bZIP member, AtbZIP72, was incorporated into the I subgroup based on our phylogenetic tree. The newly added Arabidopsis bZIP family members AT1G58110 and AT4G06598 were found to belong to the E subgroup based on the similar distribution of conserved motifs in the protein sequences with other E subgroup members. Similarly, the new member AT2G12940 was found to belong to the I subgroup.

The distribution of bZIP proteins in the subgroups differed among the five species (Additional file 2: Figure S2), suggesting that different plant species underwent different expansions of the bZIP family. The number of bZIP proteins in *T. urartu* was found to be exactly equal to that in *Ae. tauschii* for subgroups A, B, C, D, G, H, I, U1 and U3 and differed by one for subgroups F and U2 (Additional file 2: Figure S2). Furthermore, the ratio of total identified bZIP proteins in wheat to that in *T. urartu* (or *Ae. tauschii*) was 1.86 (or 1.90). For subgroups D, G, I and U1, the ratio of total identified bZIP proteins in wheat to that in *T. urartu* (or *Ae. tauschii*) was approximately 3. Thus, we estimate that approximately 100 bZIP proteins in wheat are not represented in the current draft genome.

### Microarray analyses and identification of anther development-related *TabZIP* genes

To identify *TabZIP* genes related to anther development, we performed microarray analysis to identify differentially expressed *TabZIP* genes during the developmental period from the PMC stage to the meiosis stage of wheat anther. The microarray data included five groups of comparisons, namely, between 366A and 411A (CK), 366E and 411E (CK), 366AB and 366EF (CK), 366A and 366E (CK), and 366B and 366 F (CK). “366” and “411” referred to wheat varieties BS366 (thermosensitive genic male sterile line) and Jing411 (restorer line, male-fertile), respectively. The letters “A”, “B”, “E” and “F” represented the growth conditions of BS366 and Jing411 (A: 10 °C with a 12 h light/ 12 h dark photoperiod; B: 10°C with a 14 h light/ 10 h dark photoperiod; E: 20 °C with a 12 h light/ 12 h dark photoperiod; F: 20 °C with a 14 h light/ 10 h dark photoperiod). “A” and “B” were male-sterile conditions while “E” and “F” were male-fertile conditions for BS366. For example, in comparisons groups 366A-411A (CK), the comparison was established between BS366 and Jing411 grown under the same conditions of “A” and Jing411 was regarded as control.

**Fig. 5:**
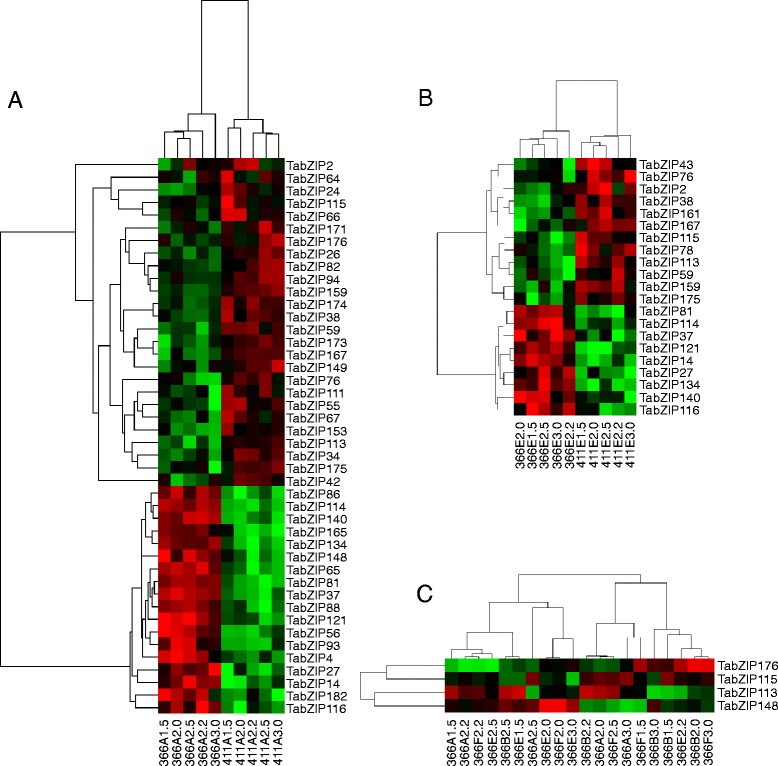
Differentially expressed genes in three comparison groups. **a** 366A-411A, (**b**) 366E-411E and (**c**) 366AB-366EF. Differentially expressed genes were defined based on *p*-values and adjusted p-values (*p* < 0.05 and adjusted *p* < 0.05). "366" and "411" represented wheat varieties "BS366" and "Jing411". The letters "A", "B", "E" and "F" referred to the grown conditions (A: 10 °C,12 h light/12 h dark photoperiod; B: 10 °C,14 h/10 h photoperiod; E: 20 °C,12 h/12 h photoperiod; F: 20 °C, 14 h/10 h photoperiod). The numbers "1.5", "2.0", "2.2", "2.5" and "3.0" represented the lengths of anthers when sampling

From the variety comparison between BS366 and Jing411 grown under 10°C with a 12 h/12 h photoperiod (male-sterile conditions for BS366), 44 *TabZIP* genes were identified as differentially expressed genes (*p* < 0.05 and adjusted *p* < 0.05), including 18 upregulated and 26 downregulated genes (Fig. 5a and Additional file 1: Table S3 and Table S4 ). The 366E-411E group identified 9 upregulated and 12 downregulated *TabZIP* genes (Fig. 5b). In the 366AB-366EF group, 4 upregulated *TabZIP* genes were identified, but no downregulated genes were detected (Fig. 5c). In addition, only one upregulated *TabZIP* gene was identified from the comparison of BS366 (10 °C, 12 h/12 h) and BS366 (20 °C, 12 h/12 h).

Altogether, among all *TabZIP* genes represented on the microarray, 48 *TabZIP* genes were differentially expressed (Table 2 and Additional file 1: Table S4). It had been reported that 8 *bZIP* genes from Arabidopsis, rice and maize had an important role in floral development [16, 18, 20, 45–48]. These 8 genes included *AtbZIP14* (*FD*) [45], *AtbZIP21* (*TGA9*) [18], *AtbZIP27* (*FDP*) [46], *AtbZIP34* [16], *AtbZIP46* (*PAN*) [47], *AtbZIP65* (*TGA10*) [18], *OsABI5* [20] and maize *DLF1* [48]. So 23 candidate *bZIP* gene were further selected from 48 differentially expressed gene based on their phylogenetic relationship and conserved motifs similarity with the 8 known floral development-related *bZIP* genes. The phylogenetic relationship and conserved motifs of 23 *TabZIP* genes and 8 known floral development-related *bZIP* genes were shown in Additional file 2: Figure S3 and S4.

**Table 2:** Basic information of 48 differentially expressed *TabZIP* genes

Gene	Gene ID	CDS (bp)	PEP (aa)	pI	Mw (kD)	bZIP domain location	Chromosomal location
*TabZIP2*	Traes_1AS_94B6230FB.1	453	150	9.67	16.28	25–87	1AS
*TabZIP4*	Traes_1AL_00A8A2030.2	684	227	5.32	24.46	68–130	1AL
*TabZIP14*	Traes_2AL_3D7807781.1	396	131	10.08	14.32	54–115	2AL
*TabZIP24*	Traes_4AS_1A68F2C29.2	1215	404	9.18	43.61	323–386	4AS
*TabZIP26*	Traes_4AS_6EDD5ACF7.2	1005	334	7.87	37.25	47–96	4AS
*TabZIP27*	Traes_4AS_F9C171219.1	678	225	9.68	24.60	150–199	4AS
*TabZIP31*	Traes_5AS_2F996234C.1	897	298	5.41	32.56	104–164	5AS
*TabZIP34*	Traes_5AL_04D3E97F0.1	900	299	5.71	31.86	108–170	5AL
*TabZIP37*	Traes_5AL_39649C38E.1	951	316	8.65	34.61	133–181	5AL
*TabZIP38*	Traes_5AL_538EC4C86.1	525	174	9.69	19.81	7–64	5AL
*TabZIP42*	Traes_5AL_CB0F2D278.1	723	240	9.69	26.51	52–113	5AL
*TabZIP43*	Traes_5AL_D9A4DFF71.1	465	154	9.16	17.17	28–91	5AL
*TabZIP55*	Traes_7AL_A8CAE984E.1	921	306	6.99	34.33	153–208	7AL
*TabZIP56*	Traes_7AL_C7CF7087B.2	522	173	8.81	18.92	2–61	7AL
*TabZIP59*	Traes_1BS_A44C97E0F.1	453	150	9.38	16.21	25–87	1BS
*TabZIP64*	Traes_1BL_C5FEAF06E.1	1212	403	9.21	42.73	263–326	1BL
*TabZIP65*	Traes_1BL_DE2CF9613.1	1440	479	9.21	51.67	407–461	1BL
*TabZIP66*	Traes_2BS_169BEF991.2	2079	692	9.04	73.71	242–305	2BS
*TabZIP67*	Traes_2BS_1B0F27580.2	951	316	9.28	35.76	13–65	2BS
*TabZIP76*	Traes_2BL_D0D6F6846.1	552	183	10.14	20.42	93–153	2BL
*TabZIP78*	Traes_3B_302AD07C6.1	807	268	5.88	29.09	141–203	3B
*TabZIP81*	Traes_3B_54638B20B.1	450	149	8.41	15.95	25–87	3B
*TabZIP82*	Traes_3B_5CABEBCE5.2	999	332	7.79	36.92	43–92	3B
*TabZIP86*	Traes_3B_A796206A0.2	1167	388	6.63	41.78	302–367	3B
*TabZIP88*	Traes_4BS_B7A8F8CD8.1	1092	363	6.57	40.33	55–102	4BS
*TabZIP93*	Traes_4BL_9422485B3.2	1128	375	6.91	40.50	177–238	4BL
*TabZIP94*	Traes_4BL_DAFEC95DD.1	1005	334	7.87	37.27	47–96	4BL
*TabZIP111*	Traes_5BL_DE53199D3.3	1098	365	8.57	39.08	284–344	5BL
*TabZIP113*	Traes_5BL_F367A99A7.1	858	285	5.63	30.39	94–156	5BL
*TabZIP114*	Traes_5BL_FB4EDEA83.2	972	323	8.31	36.00	249–307	5BL
*TabZIP115*	Traes_6BS_05264DAEA.2	1053	350	6.17	36.85	250–313	6BS
*TabZIP116*	Traes_6BS_65CDD608F.2	693	230	10.1	25.28	136–184	6BS
*TabZIP121*	Traes_7BL_625F55A12.1	438	145	10.02	16.41	1–54	7BL
*TabZIP134*	Traes_2DL_1F0CDB1CE1.1	396	131	10.08	14.34	54–115	2DL
*TabZIP140*	Traes_3DL_A71427183.1	1314	437	6.48	48.13	148–198	3DL
*TabZIP148*	Traes_4DL_F38ED7FB6.1	672	223	9.93	24.52	148–197	4DL
*TabZIP149*	Traes_5DS_011851EE7.1	474	157	9.02	17.41	27–89	5DS
*TabZIP153*	Traes_5DL_1950C1FC2.2	1578	525	6.63	58.68	205–252	5DL
*TabZIP159*	Traes_5DL_743B870D9.1	855	284	5.63	30.19	94–156	5DL
*TabZIP161*	Traes_5DL_841444545.1	555	184	9.78	20.83	13–74	5DL
*TabZIP165*	Traes_5DL_C794C1EEA.3	1101	366	6.47	39.25	269–332	5DL
*TabZIP167*	Traes_6DS_273430303.2	942	313	5.42	32.96	135–194	6DS
*TabZIP171*	Traes_6DL_F7015CE89.2	528	175	10.52	19.29	91–137	6DL
*TabZIP173*	Traes_7DS_47B5A7FFF.1	630	209	10.36	23.24	13–74	7DS
*TabZIP174*	Traes_7DS_C6A3C10A6.1	453	150	10.2	16.78	68–131	7DS
*TabZIP175*	Traes_7DS_E5A18AAA4.1	717	238	6.76	26.34	70–119	7DS
*TabZIP176*	Traes_7DL_3CE000E38.1	729	242	5.04	26.60	79–142	7DL
*TabZIP182*	Traes_7DL_EECCC4DBF.1	495	164	9.42	18.04	91–153	7DL

### Analysis of gene structures and *cis*-acting regulatory elements

**Fig. 6:**
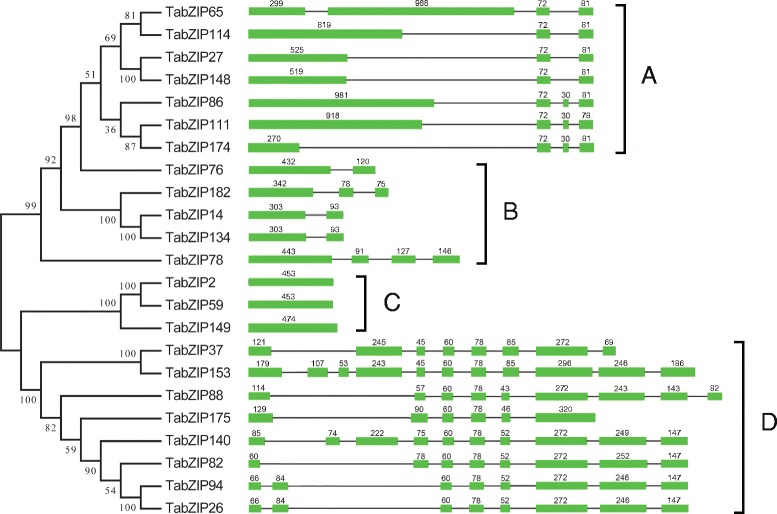
Phylogenetic analysis (left) and exon-intron structures (right) of 23 *TabZIP* genes. (Left) The phylogenetic analysis was performed using the protein sequences of 23 *TabZIP* genes. The unrooted phylogenetic tree was generated by the Neighbor-joining method and displayed using MEGA 5.0 software. Numbers above or below branches of the tree indicated bootstrap values. (Right) Only the exons, represented by green boxes, were drawn to scale. Black lines connecting two exons represented introns. Numbers above the exons of each gene structure represented the size of exons. The phylogenetic tree was separated into four subdivisions (**a**), (**b**), (**c**) and (**d**) based on the similarity of gene structures

First, we mapped the exon/intron organization of the 23 *TabZIP* genes (Fig. 6). We found that 3 genes were intronless and that the numbers of introns for the other *TabZIP* genes varied from 1 to 10. Based on the method described above, we have identified several pairs of in-paralogs within these 23 *TabZIP* genes (Additional file 1: Table S5). As shown in Fig. 6, the in-paralogs tended to share an identical or similar exon-intron composition. Three in-paralog pairs (namely, *TabZIP2* and *TabZIP59*, *TabZIP14* and *TabZIP134*, as well as *TabZIP26* and *TabZIP94*) had preserved identical gene structure organizations, while another in-paralog pair (*TabZIP27* and *TabZIP148*) had a highly constant exon-intron composition except for differing by six nucleotides within the first exon. Gene structure analysis revealed that genes with a close evolutionary relationship tended to share similar gene structures. Divergent exon lengths within the coding sequences of several *TabZIP* genes could potentially lead to the generation of functionally distinct paralogs.

**Fig. 7:**
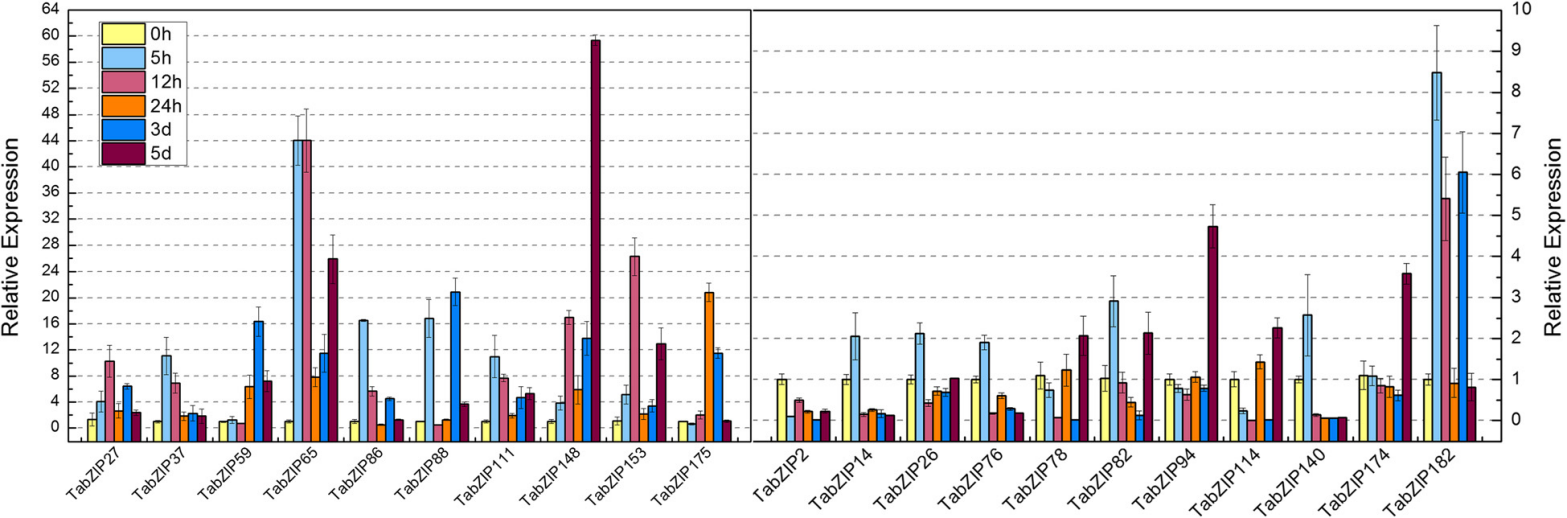
Expression levels of *TabZIP* genes in anther of wheat line BS366 following low temperature treatment. The 2^−*ΔΔC*^_T_ method was used to calculate the relative expression levels of the target genes. The expression of each *TabZIP* gene at 0 h was regarded as a reference, and other values represented the expression levels relative to the reference. Mean values and SDs were obtained from three biological replicates. (Left) 10 *TabZIP* genes (for each of them, the highest expression level after treatment was more than 10 times over the untreated control); (Right) 11 *TabZIP* genes (for each of them, the highest expression level after treatment was less than 10 times over the untreated control)

In addition, we analyzed the *cis*-acting elements in the promoter sequences of the selected *TabZIP* genes (Additional file 1: Table S6 and Table S7). Promoter sequences were available for 20 among 23 selected *TabZIP* genes. And according to prediction of cis-acting elements, 15 of these 20 *TabZIP* genes (75 %) contained LTR elements in their promoter regions, which was consistent with their expression patterns in response to low temperature (Fig. 7). Expression patterns under low temperature showed that 14 of these 15 *TabZIP* genes (93 %, except *TabZIP134*) were responsive to low temperature to varying degrees (Fig. 7). Among them, *TabZIP148* was predicted to have the most LTR elements in its promoter region, and expression analysis demonstrated that *TabZIP148* exhibited the most remarkable change in expression level after treatment because the highest expression value was over 59 times higher than the lowest value (Fig. 7).

### Expression patterns of *TabZIP* genes in response to low temperature treatment

First, 23 genes were selected from differentially expressed genes based on microarray analyses, indicating these 23 genes were more likely to be involved in anther development. Additionally, the male fertility of BS366 was controlled by temperature. Exposing to 10 °C for 5d (starting at the PMC stage and continuing to the meiosis stage) leaded to complete male sterility in BS366 [31]. Thus, we speculate that expression of genes involved in male fertility of BS366 is supposed to be affected by low temperature.

To investigate the expression patterns of *TabZIP* genes in response to low temperature (10 °C) treatment, quantitative real-time PCR (qRT-PCR) was performed using BS366 anthers that were collected at different time points from the PMC to the meiosis stage as samples. Results showed that the expressions of 21 among the 23 *TabZIP* genes were obviously responsive to low temperature and these genes exhibited complicated expression changes after different durations of treatment (Fig. 7).

Clear changes in expression values were observed for these 21 *TabZIP* genes post-treatment. The transcript levels of six of the 21 *TabZIP* genes were upregulated at each time point after treatment compared with the untreated control. *TabZIP148* exhibited the most remarkable change in expression after treatment because the highest expression value was over 59 times higher than the lowest value. *TabZIP153* and *TabZIP148* shared a similar changing tendency that transcripts increased gradually and reached a maximum at 12 h, then declined to a relatively lower level at 24 h, followed by an increase throughout the following durations.

### Tissue-specific expression patterns of *TabZIP* genes in the male-sterile line, restorer line and their F1 hybrid

Among TY806, BS366 and F1 (TY806 × BS366) grown under local environmental conditions of Fuyang (Anhui Province, China), no differences in the morphology or structure of the stamens and pistils were observed before blooming (Additional file 2: Figure S5). However, after blooming, the anthers of BS366 failed to dehisce as those of TY806 and F1, and a few pollen grains formed and were devoid of starch (Additional file 2: Figure S5). The results of pollen iodine staining demonstrated that the pollen grains of BS366 were small, irregularly shaped, nearly transparent and easily broken compared with those of TY806 and F1. Statistical analysis showed that 95.9 % of the pollen grains in TY806 and 87.0 % in F1 were circular, opaque and dark brown-black after iodine staining, while BS366 exhibited a highly male-sterile type because no Type A pollen grains were present and because 80.6 % of the pollen grains that were present were circular, opaque or partially transparent and light brown-black after iodine staining (Additional file 2: Figure S5).

**Fig. 8:**
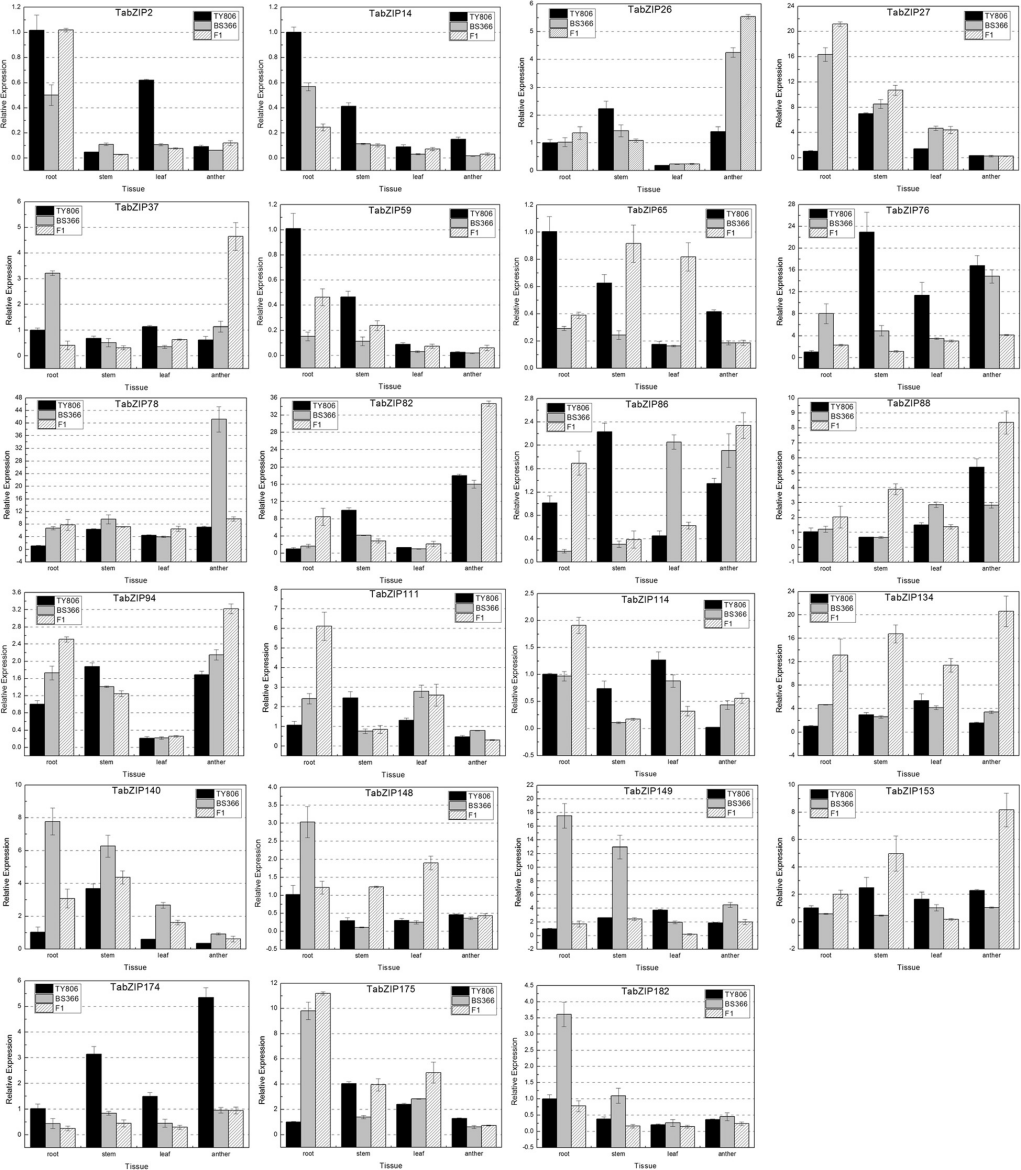
Tissue-specific expression pattern of 23 *TabZIP* genes in BS366, TY806 and their hybrid F1. BS366 is the male-sterile line and TY806 is the restorer line. The 2^−*ΔΔC*^_T_ method was used to calculate the relative expression levels of the target genes. The expression of each *TabZIP* gene at 0 h was regarded as a reference, and other values represented the expression levels relative to the reference. Mean values and SDs were obtained from three biological replicates

To explore tissue-specific and variety-specific expression pattern of the 23 *TabZIP* genes in the wheat cross combination TY806 × BS366, we obtained samples from the roots, stems, leaves and anthers of TY806, BS366 and their hybrid F1 at the meiosis stage to analyze their expression patterns. The results revealed that every *TabZIP* gene exhibited a distinct tissue-specific expression pattern in TY806, BS366 and their hybrid F1 at the meiosis stage (Fig. 8). Additionally, as in-paralogs, both the *TabZIP2/TabZIP59* and *TabZIP26/TabZIP94* pairs exhibited similar tissue expression patterns, while *TabZIP14* showed a different expression profile compared to its in-paralog *TabZIP134.* Furthermore, *TabZIP27* and *TabZIP148* were both expressed at low levels in anther tissue (Fig. 8).

Among the 23 *TabZIP* genes, 11 were predominantly expressed in the anther, which indicated that they were more likely to have a role in anther development during meiosis.

**Fig. 9:**
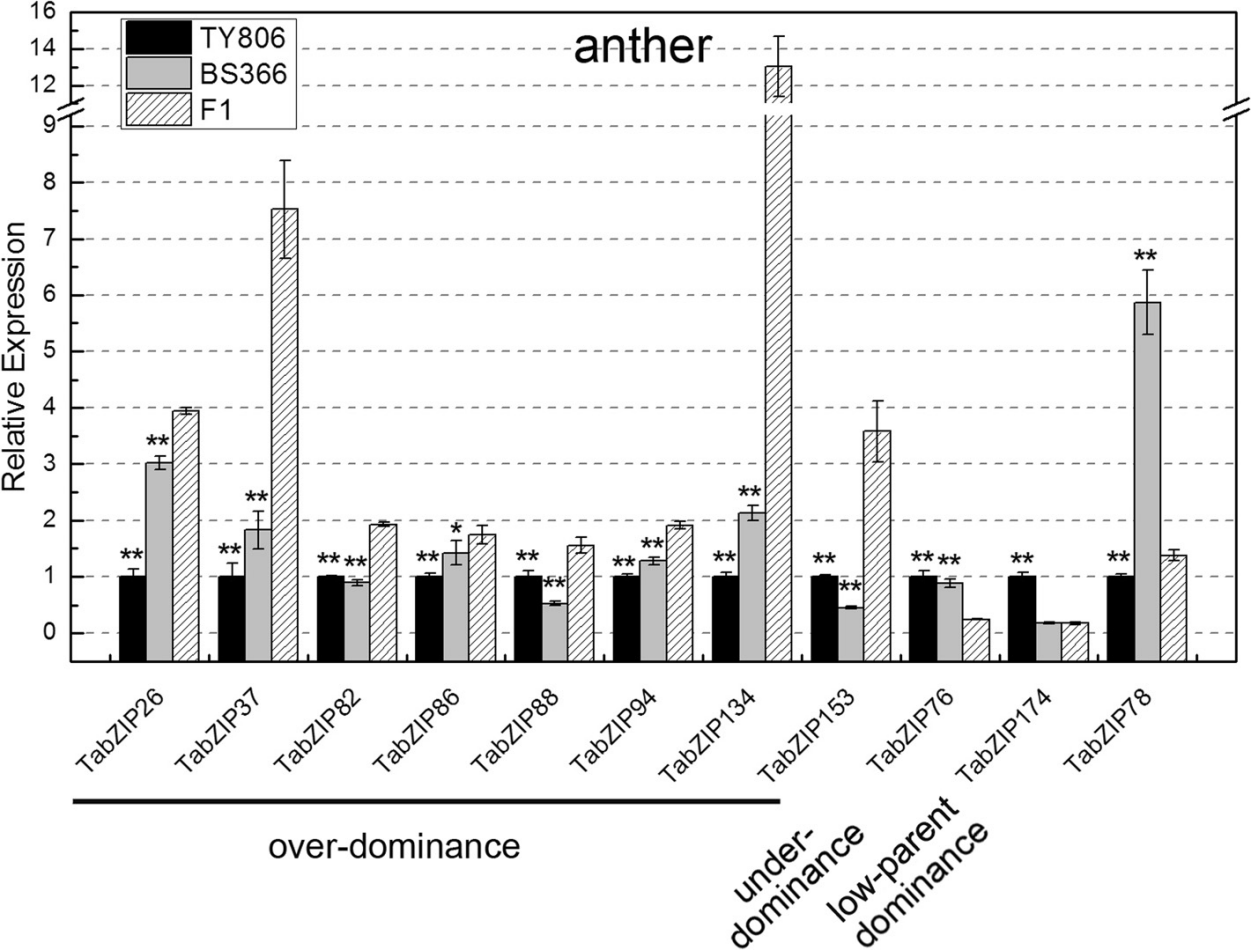
Differential expression of 11 *TabZIP* genes in anther of TY806, BS366 and their hybrid F1. These 11 *TabZIP* genes were predominantly expressed in anther. The expression of each gene in anther of TY806 was regarded as a reference, and other values represented the expression levels of this gene in anther of BS366 and F1 relative to the reference. Single asterisk indicated the expression difference between TY806 and F1 or between BS366 and F1 was significant at *p* < 0.05 by the *t*-test and double asterisks meant a highly significant difference between TY806 and F1 or between BS366 and F1. These 11 *TabZIP* genes were sorted into three distinct gene expression modes based on their deviation from the mid-parent prediction: over-dominance, under-dominance and low-parent dominance. *TabZIP78* did not belong to any of four kinds of modes (over-dominance, under-dominance, low-parent dominance and high-parent dominance)

Furthermore, we selected the anther expression data of these 11 *TabZIP* genes to analyze the expression differences among the three wheat lines (Fig. 9). Significant differences between the F1 and TY806 or between the F1 and BS366 were assessed using a *t*-test. These 11 *TabZIP* genes, excluding *TabZIP78*, were further classified into three distinct gene expression modes based on their deviation from the mid-parent prediction: over-dominance, under-dominance and low-parent dominance [49]. The high-parent dominant genes and low-parent dominant genes were defined according to the following criterion that the expression level in F1 genotype was significantly different from that in one parent and no significantly different from that in another parent. And the genes were identified as over-dominance or under-dominance when the expression level in F1 genotype was significantly higher or lower than those in both inbred parents [49]. *TabZIP134* displayed such extremely clear over-dominance that the expression level in the F1 was approximately 6-fold greater than that in BS366, while the corresponding multiple for *TabZIP37* was approximately 4-fold. Among the genes with over-dominance mode, *TabZIP26*, *TabZIP37*, *TabZIP86*, *TabZIP94* and *TabZIP134* showed similar expression trend because they were expressed maximally in the F1 and had the lowest expression level in TY806. *TabZIP76* showed an under-dominance mode because the slight expression in the F1 was significantly lower than those in TY806 and BS366. *TabZIP174* was expressed primarily in TY806 and exhibited slight expression in both BS366 and F1, which belonged to the low-parent dominance mode. In conclusion, the *TabZIP* genes showed multiple gene expression modes in TY806, BS366 and their F1 hybrid, and these results were consistent with relevant studies in maize and rice that supported the theory that multiple modes of gene action collaboratively contributed to heterosis [49, 50].

**Fig. 10:**
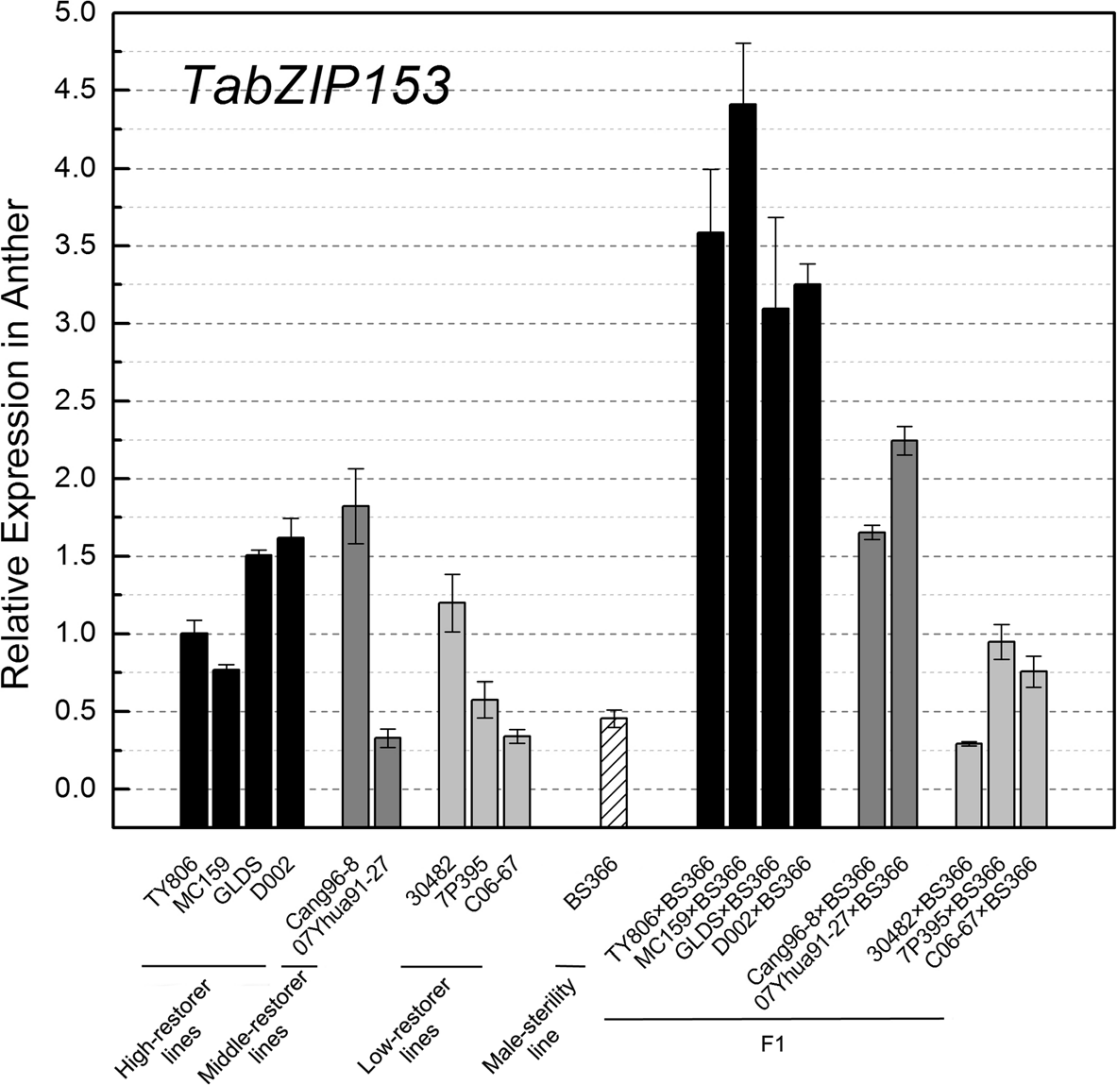
Expression profiles of *TabZIP153* in anther of 9 groups of two-line hybrid combinations. Wheat varieties TY806, MC159, GLDS and D002 are 4 high-restorer lines, Cang96-8 and 07Yhua91-27 are middle-restorer lines and 30482, 7P395 and C06-67 are low-restorer lines. BS366 is male-sterility line. F1s are hybrids between BS366 and the corresponding restorer lines. Anthers were harvested from the corresponding wheat plants 5d after the flag leaf had half-emerged from the collar of the penultimate leaf

*TabZIP26*, *TabZIP37*, *TabZIP134* and *TabZIP153* exhibited significant over-dominance expression modes (Fig. 9). To further verify these different expression modes in other two-line hybrid combinations, we selected other 8 groups of two-line hybrid combinations as experimental materials (Fig. 10). All 9 restorer lines used for our study could be classified into three categories based on their male-restoring ability: 4 high-restorer lines (TY806, MC159, GLDS, D002), 2 middle-restorer lines (Cang96-8, 07Yhua91-27) and 3 low-restorer lines (30482,7P395, C06-67). Results showed that *TabZIP153* exhibited over-dominance expression in the cross combinations of 4 high-restorer lines and 07Yhua91-27, 7P395 and C06-67, whereas it exhibited under-dominance expression in the 30482 × BS366. Thus, *TabZIP153* exhibited over-dominance in 7 among 9 groups of cross combinations.

## Discussion

### Evolutionary relationship of wheat, *T. urartu*, *Ae. tauschii*, barley and *Brachypodium*

The evolutionary relationships between rice, *Brachypodium*, barley and wheat have been speculated based on the mean synonymous substitution rates (Ks) of orthologous gene pairs [32]. Wheat shared a common ancestor with rice approximately 40–54 million years BC, and the divergence time of *Brachypodium* from other gramineous plants was approximately 32–39 million years BC. Subsequently, barley diverged from other gramineous plants approximately 3–4 million years BC [32].

Wheat derived from hybridization between the cultivated tetraploid emmer wheat (*T.* dicoccoides, AABB) and diploid goat grass (*Ae. tauschii*, DD) approximately 8,000 years ago [21]. The three diploid progenitor genomes of wheat, AA from *T. urartu*, BB from a species that might be from the section *Sitopsis*, and DD from *Ae. tauschii*, originated from a common *Triticeae* ancestor approximately 2.5-4.5 million years ago [21].

Notably, *T. urartu*, *Ae. tauschii* and barley, all of which were diploid, shared the same chromosome number (seven), suggesting that they had a relatively close evolutionary relationship. Furthermore, synteny block identification among *Brachypodium*, barley and *Ae. Tauschii* revealed that they were most likely to share a common ancestral set of five chromosomes [32]. The draft genome sequence of *Ae. tauschii* revealed 11,289 orthologous gene pairs between barley and *Ae. tauschii* and 14,675 between *Brachypodium* and *Ae. tauschii* [22]. Although the *T. urartu* genome was more than 18 times larger than that of *B. distachyon*, the average gene sizes of these two organisms were similar, and the predicted gene number (34,879) in the *T. urartu* genome was only approximately 1.37-fold that of *B. distachyon* (25,532) [23]. In conclusion, previous studies have demonstrated that wheat, *T. urartu*, *Ae. tauschii*, barley and *Brachypodium* are evolutionarily close in the Gramineae family. However, the real evolutionary relationship among them still remains mysterious.

bZIP family genes appeared before the divergence between monocots and dicots and the structure and function of most *bZIP* genes most likely remained conserved during angiosperm evolution [2]. bZIP transcription factors were associated with the evolution of plants [51]. Thus, in this study, we deduced the possible evolutionary relationship among them based on orthology analyses of bZIP transcription factors. In the case of the bZIP family, A sub-genome of wheat was more similar with *T.urartu* than other three genomes (34.5 % of bZIP family members in A sub-genome of wheat were orthologous to *T.urartu*). Similarly, among *T.urartu*, *Ae.tauschii*, barley and *Brachypodium*, D sub-genome of wheat was most similar with *Ae.tauschii* (42.6 % of bZIP family members in D sub-genome of wheat were orthologous to *Ae.tauschii*). However, B sub-genome of wheat was more similar with barley (49.2 %) and *Brachypodium* (47.6 %) than *T.urartu* (30.2 %) and *Ae.tauschii* (30.2 %) (Additional file 2: Figure S6). In addition, it was observed from Fig. 3 that wheat had a closer evolutionary relationship with barley and *Brachypodium* than *T. urartu* and *Ae. tauschii*. Approximately 8,000 years ago, wheat arose as a result of hybridization between the cultivated tetraploid emmer wheat (*T. dicoccoides*, AABB) and diploid goat grass (*Ae. tauschii*, DD) [21]. It could be deduced that much more than approximately 8,000 years ago, *T. dicoccoides* (AABB) was generated from the hybridization between *T. urartu* (donor of A sub-genome) and the donor of B sub-genome. So *T. urartu* and *Ae. tauschii* were very old species. From speciation, wheat had undergone at least 8,000 years of evolution course. And after long-term natural selection and artificial domestication and cultivation, A and D sub-genomes of modern wheat were no more what they had been. If the self-evolution of *T. urartu* and *Ae .tauschii* was taken into consideration, there might exist bigger differences between A sub-genome (or D sub-genome) of modern wheat and modern *T. urartu* (or *Ae. tauschii*). However, it was a generally acknowledged fact that barley was one of close relatives of wheat, and *Brachypodium* had been regarded as a suitable model system for studies on temperate cereals, such as wheat, because of its small and similar genome [52]. So it was understandable that wheat had a closer phylogenetic relationship with barley and *Brachypodium* than *T. uratu* and *Ae. tauschii* that was concluded from orthology analyses. Our results provided deeper insight into evolutionary relationship of wheat and its relatives.

### The evolution of bZIP transcription factor family

The phylogenetic analysis of bZIP transcription factors in rice and Arabidopsis indicated that these genes appeared preceding the divergence between monocots and dicots [2]. In plants, the structure and function of most bZIP genes most likely maintained conserved during angiosperm evolution [2]. Indeed, the majority of OsbZIP proteins were found to be orthologous to AtbZIP proteins [2]. According to the orthologs identified in wheat and its four relatives (Table 1 and Additional file 1: Table S2), barley had 72 groups of orthologs with *Brachypodium* and 64 groups of orthologs with wheat. Notably, 69.4 % of the TubZIP proteins were orthologous to 68.8 % of the AetbZIP proteins (Fig. 1 and Additional file 2: Figure S1).

During evolution process of bZIP transcription factor family, several new subgroups or clades had come into being in the *T. urartu* and *Ae. tauschii* genomes. Based on the phylogenetic tree (Fig. 4), Subgroups A, B, C, D, E, F, G, H, I, and S, as well as U1 and U3, clearly consisted of bZIP proteins that were present in all five species, indicating that the origin of all 10 subgroups and U1 and U3 might have predated the speciation events between eudicot and monocot plants. In contrast, U2 and U4 consisted of only bZIP proteins from *T. urartu* and *Ae. tauschii* but not from wheat, Arabidopsis or rice. Previous studies had deduced that wheat and rice share a common ancestor approximately 40 to 54 million years BC [32]. The observation that U2 and U4 bZIP proteins were only present in the *T. urartu* and *Ae. tauschii* genomes suggests that these genes likely arose after the evolutionary divergence of rice from other gramineous plants; however, these genes disappeared during the evolution or domestication of wheat, which was consistent with the observation that wheat underwent genetic loss as a consequence of domestication [21].

Additionally, bZIP family went through different degrees of expansion in different gramineous plants. From Table 1, it could be deduced that wheat had many more in-paralogs than its four relatives *T. urartu*, *Ae. tauschii*, barley and *Brachypodium*. On average, each *TabZIP* gene had 0.702 in-paralogs. Two explanations likely accounted for this high average: (i) wheat was derived from two hybridizations between three diploid progenitors, and as a consequence, several copies of homologous genes had been gathered together in the wheat genome; (ii) a vast number of repetitive genes were present in the wheat genome as a result of gene duplication. By contrast, both *T. urartu* and *Ae. tauschii* had few in-paralogs in the case of the bZIP family (Fig. 1, Table 1 and Additional file 1: Table S2), indicating that gene duplication events were likely to occur very rarely within both *T. urartu* and *Ae. tauschii*, or that the gene duplication frequency was low during the long evolutionary history of at least 8,000 years because gene duplication can accelerate the generation of in-paralogs within a species [32]. Few gene duplication events might limit the expansion of a gene family such as the bZIP transcription factor family, which might account for the similar sums of bZIP family members between *T. urartu* and *Ae. tauschii*.

### Function of bZIP family genes in anther development

Previous studies have demonstrated that bZIP transcription factors participate in anther development [13–20]. In our study, we have identified 48differentially expressed *bZIP* genes from anther tissue (*p* < 0.05 and adjusted *p* < 0.05). Among them, *TabZIP113* and *TabZIP148* were identified as differentially expressed *bZIP* genes from the comparison between BS366 grown under 10 °C (male-sterile conditions) and BS366 grown under 20 °C (male-fertile conditions). *TabZIP113* (locus: JP879345) was isolated previously from pooled seedlings 8–12 days after germination and florets from pre-meiosis to immediately before anthesis [53]. In addition, *TabZIP113* shared high sequence similarity with the mRNA sequence (locus: JV912179) isolated from anthers and pre-anthesis spikes of fertile and sterile plants [54]. *TabZIP148* (locus: CK209553) had high similarity with Ta_Contig57821 (locus: JV993809) and Ta_Contig57627 (locus: JV993615), which were also isolated from anthers and pre-anthesis spikes of fertile and sterile plants [54]. These results suggested that *TabZIP113* and *TabZIP148* were possibly related to anther development.

Additionally, sequence analysis revealed that *TabZIP88* and *TabZIP175* were highly similar to Arabidopsis *TGA9* and *TGA10* in the protein sequence, which played a role in anther patterning and dehiscence [18]. And expression analysis showed that *TabZIP88* is predominantly expressed in anther (Fig. 8). Therefore, *TabZIP88* might share similar function with *TGA9* and *TGA10*. Besides, *TabZIP86*, which was also predominantly expressed in anther (Fig. 8), had complete sequence and domain identities with *OsABI5* of 61.93 % and 93 %, respectively. *OsABI5* was required for pollen development and to maintain normal fertility in rice [20].

Expression in anther demonstrated that *TabZIP* genes exhibited multiple gene expression modes in the TY806 × BS366 heterotic cross (Fig. 9). There were mainly three possibilities accounting for the differential transcription of the same gene in TY806 × BS366: (i) the interplay between promoter INDEL and regulated expression of *trans*-acting factors (such as transcription factors); (ii) microRNA regulation; (iii) DNA methylation. Previous study have revealed that sequence polymorphism within promoter alleles between inbreds preferentially occurs in those differentially transcribed genes. Promoter INDELs and differential expression of transcription factors may result in all possible modes of gene action [49]. miRNAs generally act as post-transcriptional regulators of gene expression in plants [55–57]. DNA methylation significantly contributes to gene expression pattern and the promoter-methylated genes tend to show a greater degree of tissue-specific expression [58, 59].

Compared with TY806 and F1, the most special fertility-related characters of BS366 under specific low temperature conditions were that the anthers fail to dehisce normally and that few pollen grains were produced and devoid of starch. As shown in Fig. 9, *TabZIP78* was expressed predominantly in anthers of BS366 relative to those of TY806 and their F1 hybrid under low temperature conditions (10 °C). Furthermore, *TabZIP78* was highly homologous to *AtbZIP34*, which was involved in the control of several metabolic pathways of developing pollen in Arabidopsis, including pollen wall patterning [16]. Thus, *TabZIP78* was probably involved in anther development, however, whether *TabZIP78* had a correlation with the character of male sterility in BS366 required further functional verification.

## Conclusions

In this study, 187 bZIP family genes were comprehensively identified from the current wheat genome. 98, 96 and 107 members of bZIP family were also identified from the genomes of *T.urartu*, *Ae.tauschii* and barley, respectively. The orthologs and in-paralogs between each pair of wheat and its relatives had been also identified. Results showed *T.urartu* and *Ae.tauschii* were evolutionarily close and wheat probably had a closer phylogenetic relationship with barley and *Brachypodium* than *T.urartu* and *Ae.tauschii*. Based on microarray data, 48 differentially expressed *TabZIP* genes were identified to be related to anther development from the comparison between the male-sterile line and the male-fertile line. Genes with close evolutionary relationship tended to share similar gene structures. Expression of 21 among 23 selected *TabZIP* genes were obviously responsive to low temperature. These 23 *TabZIP* genes all exhibited distinct tissue-specific expression pattern. Among them, 11 *TabZIP* genes were predominantly expressed in anther and most of them showed over-dominance expression mode in the cross combination TY806 × BS366. This study provided a comprehensive overview of the bZIP family in wheat and its relatives and laid a foundation for further evolution research and functional characterization of anther development related *TabZIP* gene.

## Methods

### Identification of bZIP transcription factors in wheat, *T. urartu*, *Ae. tauschii* and barley

To identify putative bZIP transcription factor genes in wheat, *T. urartu*, *Ae. tauschii* and barley, HMM searches of all annotated proteins in these five genomes were conducted using the HMM profiles as queries with the hmmsearch program, which is included in the HMMER software package [60]. Wheat genomic data were downloaded from the web site Gramene (Release note 41, http://www.gramene.org/) [61]. *T. urartu* and *Ae. tauschii* genomic data were downloaded from the web site http://gigadb.org/ [62]. Barley genomic data was downloaded from the web sites ftp://ftpmips.helmholtz-muenchen.de/plants/barley/. The HMM profiles of the bZIP domain (PF00170 and PF07716) were extracted from the Pfam database (http://pfam.sanger.ac.uk/) [63]. And the HMM search was performed using an E-value cutoff < 1.0. The contig sequences were removed, and the remaining protein sequences were verified for the presence of the typical bZIP domain using Pfam with an E-value cutoff < 1.0.

### Identification of orthologs and in-paralogs between wheat and other species

Orthologs and in-paralogs between any pair of five species wheat, *T. urartu*, *Ae. tauschii*, barley and *Brachypodium* were identified using Inparanoid 7 software (http://inparanoid.sbc.su.se/cgi-bin/index.cgi) [40]. Two user-defined criteria were applied to each pairwise match: (1) an all-against-all blast score cutoff of 50 bits and (2) an overlap cutoff of 50 %, namely, the matching region must exceed 50 % of the longer sequence in length. Furthermore, a confidence value was defined to show the similarity of each in-paralog to the primary ortholog in the same species on a scale ranging from 0 to 100 % (Additional file 1: Table S2) [40]. A greater confidence value indicated that the in-paralog was more similar to the primary ortholog compared with the other in-paralogs. The orthology figures were generated using a self-writing Perl program employing the SVG module.

### Multiple sequence alignment and phylogenetic analysis of bZIP proteins

Multiple sequence alignment of the identified TabZIP, TubZIP, AetbZIP proteins was conducted using the ClustalX program (Version 2.1) with default settings [64]. Meanwhile, 76 Arabidopsis and 94 rice bZIP family members were added to the phylogenetic analyses to determine the classification of TabZIP, TubZIP and AetbZIP proteins because Arabidopsis and rice were model plants for eudicots and monocots, respectively [1, 2]. The unrooted tree was generated using ClustalX and displayed by MEGA 5.0 [65].

### Microarray analyses

The microarray analyses was primarily based on our previous original Affymetrix microarray data. Samples were collected from wheat cv. Jing411 and BS366 of different developmental stages and under different treatment conditions (10 °C with 12 h light/12 h dark, 10 °C with 14 h light/10 h dark, 20 °C with 12 h light/12 h dark and 20 °C with 14 h light/10 h dark). The operating procedures of the whole experimental process were detailedly described in our previous study [31]. For microarray data analysis, the image (cel) files were imported into the online tool Babelomics 4.3 (http://babelomics.bioinfo.cipf.es/) [66]. Subsequently, data normalization was performed using RMA methods, and expression differentiation analysis was conducted using limma methods. Five comparison groups were established: 366A-411A, 366E-411E, 366AB-366EF, 366A-366E and 366B-366 F. “366” represented wheat variety BS366 and “411” represented wheat variety Jing411. The letters “A”, “B”, “E” and “F” referred the growth conditions of BS366 and Jing411 (A: 10 °C with a 12 h light/ 12 h dark photoperiod; B: 10 °C with a 14 h light/ 10 h dark photoperiod; E: 20 °C with a 12 h light/ 12 h dark photoperiod; F: 20 °C with a 14 h light/ 10 h dark photoperiod). And the sampling timepoints were detailed in Additional file 1: Table S8.

Probe sets that were up- or downregulated with *p* < 0.05 and adjusted *p* < 0.05 in all comparison groups compared to the corresponding control were considered to be differentially expressed. Nucleic acid and amino acid sequence alignments were performed to establish the corresponding relationship between probe sets and 108,569 wheat genes from the wheat genomic data at the Gramene web site. A probe set was determined to represent one wheat gene using BLASTn and BLASTx tools in accordance with the following criteria: (1) an alignment parameter E-value ≤ 1e-5 between the target sequence of the probe set and CDS sequence of a wheat gene and (2) an alignment parameter E-value ≤ 1e-3 for the protein sequences between the target of the probe set and this gene. The heatmaps were generated with Cluster 3.0 and displayed by Java Treeview.

### Analysis of protein motifs, gene structures and *cis*-acting regulatory elements

The protein sequences of 23 *TabZIP* genes were searched for common conserved motifs using the MEME analysis tool (version 4.9.1, http://meme-suite.org/tools/meme) [67], and a limit of 15 motifs was specified with all other parameters set to default. To obtain information regarding the intron and exon structure, the CDS sequences of these 23 *bZIP* genes were aligned with their corresponding genomic sequences using GSDS (http://gsds.cbi.pku.edu.cn/index.php) [68]. To analyze their promoter regions, the 1.5-kb promoter regions were selected and screened against the PlantCARE database (http://bioinformatics.psb.ugent.be/webtools/plantcare/html/) [69].

### Sample collection for morphological observation and pollen iodine staining

To observe the phenotype differences in floral organs among Jing411, TY806, BS366 and the F1 (TY806/BS366) grown under natural male-sterile conditions of Fuyang, Anhui Province, approximately 60 reproductive organs (each including a pistil and three stamens) were dissected from 60 spikelets, which were collected from 20 primary stems for each wheat line 3 days before blooming. Additionally, approximately 150 anthers were excised from 50 spikelets that were collected from 15 primary stems with small tweezers for each wheat line 1 day after blooming. The representative reproductive organs and anthers were imaged using a Leica MZ16F stereomicroscope (Leica Microsystems, Wetzlar, Germany).

Thirty anthers from each wheat line were used for pollen iodine staining, and the procedures were as follows. (i) A mature anther was positioned on the slide, and a drop of distilled water was placed on the anther. (ii) The anther was squeezed using small tweezers to release the pollen grains. (iii) Then, 1 to 2 drops of I_2_-KI solution [6.7‰ (g/ml) KI and 3.3‰ (g/ml) I_2_] were added, and a coverslip was mounted on the sample. (iv) The pollen grains were observed after iodine staining, and images were obtained using an Olympus BX41 laboratory microscope (Olympus, Tokoyo, Japan). Based on the morphology of the pollen grains after iodine staining, they were classified into 4 types: (A) circular, opaque and dark brown-black; (B) circular, opaque or partially transparent and light brown-black; (C) circular, transparent and light yellow; or (D) transparent with an irregular shape and light yellow. The number of pollen grains in each classification type was counted, and the percentage of each type for the 4 wheat lines was calculated.

### Plant materials and treatments

For the low-temperature treatment experiment, the wheat TGMS line BS366 were grown in the soil in plastic pots buried in the field. The wheat plants underwent vernalization naturally in the field. Low-temperature treatment was initiated when the flag leaf had half-emerged from the collar of the penultimate leaf (about 1.5 mm in anther length). Plants of uniform size were selected and transferred to the phytotron (Conviron, Winnipeg, Canada) and continue to grow at 10°C with a 12-h light/12-h dark photoperiod for 5d. During this period, anthers were harvested at 0 h, 5 h, 12 h, 24 h, 3d and 5d post-treatment with home-made dissecting needles under the dissecting microscope (Olympus SZX12, Tokoyo, Japan) and used for the expression analysis. To maximize the sampling consistency, about 30 anthers were separated from 10 spikelets, which were collected from 3 primary stems at each sampling timepoint.

For tissue-specific expression analyses of 23 *TabZIP* genes in the wheat cross combination TY806 × BS366, plant materials were collected from the wheat varieties TY806, BS366 and their F1 hybrid grown in the field in Fuyang, Anhui Province, where BS366 was a male-sterile variety, and TY806 and F1 were male-fertile varieties. The roots, stems, leaves and anthers of the three varieties were harvested 5d after the flag leaf had half-emerged from the collar of the penultimate leaf and three biological replicates were harvested for each sample.

To analyze expression profiles of *TabZIP153* in anther of 9 groups of two-line hybrid combinations, anthers were harvested from 18 wheat varieties at the same developmental stage as described above. These 18 wheat varieties included TY806, MC159, GLDS, D002, Cang96-8, 07Yhua91-27, 30482, 7P395, C06-67 and their hybrids with BS366.

After sampling, all plant materials used for the above studies were frozen immediately in liquid nitrogen and stored at −80 °C.

### RNA extraction and qRT-PCR

For the expression analysis of the *bZIP* genes, total RNA was extracted from plant tissues using TRIzol reagent (Ambion, USA) according to the manufacturer’s instructions. First-strand cDNA synthesis was performed using a TaKaRa PrimeScript™ RT Reagent Kit with gDNA Eraser (TaKaRa, Dalian, China). qRT-PCR analysis was conducted using an Eco Real-Time PCR system (Illumina, San Diego, CA, USA) with TaKaRa SYBR® Premix Ex Taq™ (Tli RNase H Plus) (TaKaRa, Dalian, China). Wheat *Actin* (Gene ID: 542814) served as an internal control for the expression studies.

The primers for all genes were designed using the Primer Premier 5.0 program [70]. Each reaction was performed in triplicate in a reaction volume of 10 μl. The qRT-PCR parameters were as follows: 95 °C for 30 s, 40 cycles of 95 °C for 5 s and 55 °C for 30 s. For the melting curve analysis, a program of 95 °C for 15 s followed by a constant increase from 55 °C to 95 °C was included after the PCR cycles. The expression analysis of the *bZIP* genes and wheat *actin* gene was performed using the same PCR procedure as detailed above or with a slightly adjusted annealing temperature. The relative gene expression levels were analyzed according to the ${2}^{-\varDelta \varDelta {C}_T}$ method [71].

### Availability of supporting data

All the supporting data is included within the article and its additional files.

## Additional files

Additional file 1:
**Table S1.** Nomenclature and chromosomal location of bZIP family genes in wheat, *T.urartu*, *Ae.tauschii*, barley and *Brachypodium.* **Table S2.** Orthologs and in-paralogs in bZIP family between each pair of wheat and its relatives. **Table S3.** Numbers of differentially expressed *bZIP* genes in five comparison groups. **Table S4.** Lists of differentially expressed *bZIP* genes in five comparison groups. **Table S5.** In-paralog pairs within 23 TabZIP proteins we studied. **Table S6.** Distribution of *cis*-acting elements in promoter regions of *TabZIP* genes. **Table S7.** Functions of *cis*-acting elements in promoter regions of *TabZIP* genes. **Table S8.** Wheat lines, growth conditions, and sampling timepoints for microarray data analysis. The sample features were represented by the wheat line name and a letter (A, B, E or F) followed by a number referring to the anther length when samples were taken. The letters ‘A’, ‘B’, ‘E’ and ‘F’ indicated the growth conditions of wheat lines. ‘A’: 10 °C with a 12 h-light/12 h-dark photoperiod; ‘B’: 10 °C with a 14 h-light/10 h-dark photoperiod; ‘E’: 20 °C with a 12 h-light/12 h-dark photoperiod; ‘F’: 20 °C with a 14 h-light/10 h-dark photoperiod. (ZIP 146 kb) Additional file 2:
**Figure S1.** Orthologous *bZIP* genes between *T.urartu* and *Ae.tauschii.* **Figure S2.** Distribution of bZIP family genes in subgroups and clades. bZIP family genes of wheat, *T.urantu*, *Ae.tauschii*, rice and Arabidopsis were divided into 10 subgroups and 4 clades. The number in the bracket indicated the total number of bZIP family genes in the corresponding genome. **Figure S3.** Phylogenetic relationship of 23 TabZIP proteins and 8 known floral development related bZIP proteins. **Figure S4.** Distribution of conserved motifs in 23 TabZIP and 8 known floral development related bZIP proteins. **Figure S5.** Morphological characteristics of floral organs and pollen iodine staining. The wheat materials were taken from Fuyang (Anhui Province), where Jing411, TY806 and F1 were male-fertile while BS366 was male-sterile. The first row showed the stamen and pistils before blooming; The second row showed anthers after blooming; The third row showed iodine staining results of pollen grains; The fourth row showed the statistical data of pollen iodine staining. The total pollen count used for data statistics was about 250 in four wheat lines. A, B, C and D meant 4 different kinds of shape-color types for pollen grains after iodine staining: (A) circular, opaque and dark brown-black; (B) circular, opaque or partially transparent and light brown-black; (C) circular, transparent and light yellow; (D) transparent in an irregular shape and light yellow. **Figure S6.** Number of bZIP family members in each genome (or sub-genome) and number of ortholog pairs between each pair of genomes and sub-genomes. Each box presented a genome or sub-genome, and the numbers in the boxes referred to the sums of bZIP family members in respective genomes. The number besides the straight line connecting each pair of genomes and sub-genomes indicated the sum of ortholog pairs between them. (ZIP 2480 kb)

## Abbreviations

bZIP: Basic leucine zipper
TF: Transcription factor
MEME: Multiple expectation maximization for motif elicitation
qRT-PCR: Quantitative real-time polymerase chain reaction
LTRE: Low temperature responsive element
TGMS: Thermo-sensitive genic male sterile
ABF: ABRE binding factor
