# Supplementary material for: Genome-wide identification and evolutionary analyses of bZIP transcription factors in wheat and its relatives and expression profiles of anther development related TabZIP genes
Source: BMC Genomics. 2015 Nov 18;16:976. doi: 10.1186/s12864-015-2196-7 (PMC4652339; doi:10.1186/s12864-015-2196-7)
Supplement: Additional file 2: — Figure S1. Orthologous bZIP genes between T.urartu and Ae.tauschii. Figure S2. Distribution of bZIP family genes in subgroups and clades. bZIP family genes of wheat, T.urantu, Ae.tauschii, rice and Arabidopsis were divided into 10 subgroups and 4 clades. The number in the bracket indicated the total number of bZIP family genes in the corresponding genome. Figure S3. Phylogenetic relationship of 23 TabZIP proteins and 8 known floral development related bZIP proteins. Figure S4. Distribution of conserved motifs in 23 TabZIP and 8 known floral development related bZIP proteins. Figure S5. Morphological characteristics of floral organs and pollen iodine staining. The wheat materials were taken from Fuyang (Anhui Province), where Jing411, TY806 and F1 were male-fertile while BS366 was male-sterile. The first row showed the stamen and pistils before blooming; The second row showed anthers after blooming; The third row showed iodine staining results of pollen grains; The fourth row showed the statistical data of pollen iodine staining. The total pollen count used for data statistics was about 250 in four wheat lines. A, B, C and D meant 4 different kinds of shape-color types for pollen grains after iodine staining: (A) circular, opaque and dark brown-black; (B) circular, opaque or partially transparent and light brown-black; (C) circular, transparent and light yellow; (D) transparent in an irregular shape and light yellow. Figure S6. Number of bZIP family members in each genome (or sub-genome) and number of ortholog pairs between each pair of genomes and sub-genomes. Each box presented a genome or sub-genome, and the numbers in the boxes referred to the sums of bZIP family members in respective genomes. The number besides the straight line connecting each pair of genomes and sub-genomes indicated the sum of ortholog pairs between them. (ZIP 2480 kb) [file 12864_2015_2196_MOESM2_ESM.zip › 12864_2015_2196_add2.zip/Additional_file_1_Fig_S5.pdf]

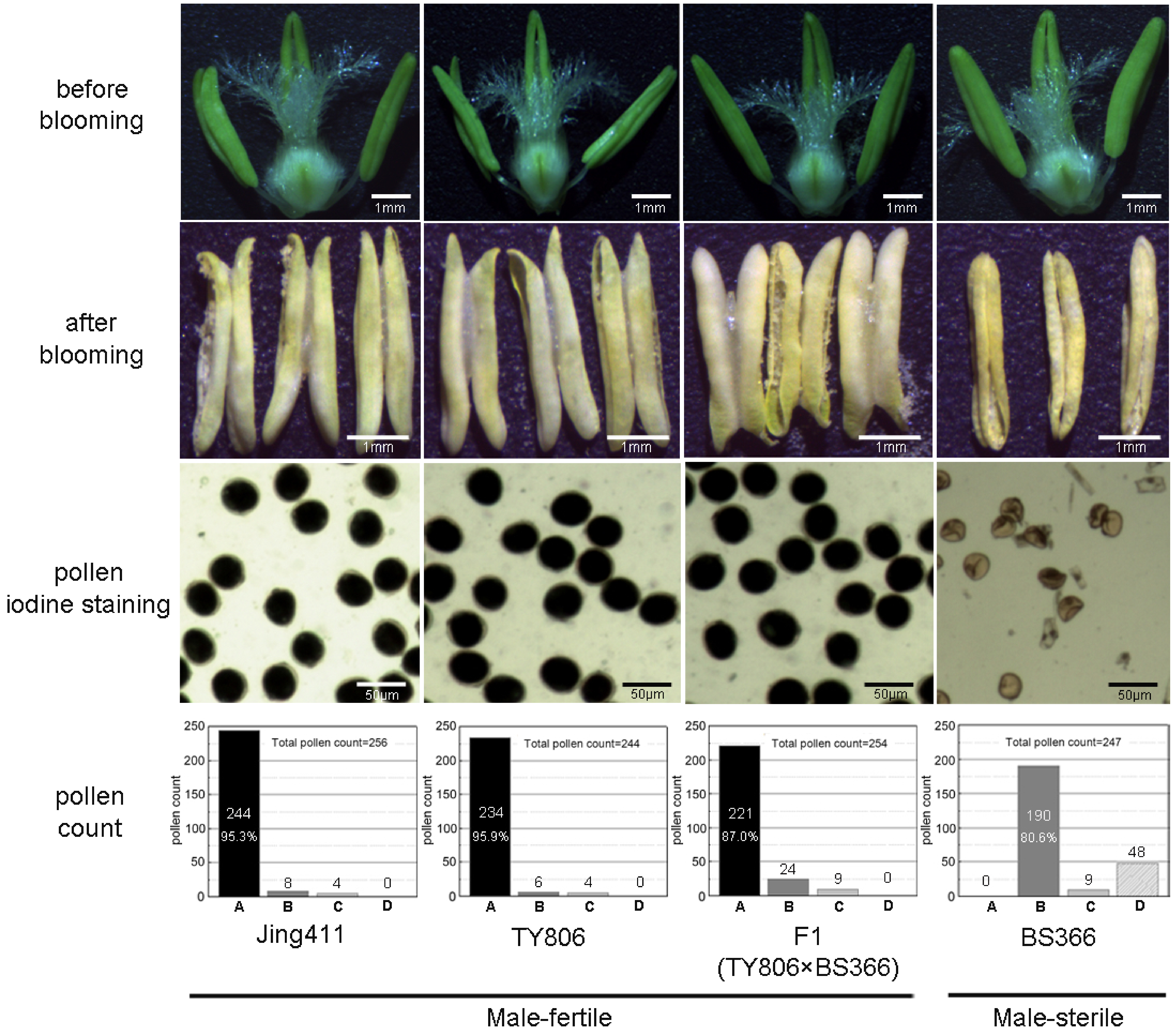
before blooming
after blooming
pollen iodine staining
pollen count
1mm
50μm
Total pollen count=256
244
95.3%
8
4
0
Total pollen count=244
234
95.9%
6
4
0
Total pollen count=254
221
87.0%
24
9
0
Total pollen count=247
0
190
80.6%
9
48
pollen count
A
B
C
D
Jing411
TY806
F1 (TY806×BS366)
BS366
Male-fertile
Male-sterile
